# Medical Students' Attitudes Toward the COVID-19 Vaccine and Medical School Vaccine Education: A Survey Study

**DOI:** 10.7759/cureus.75168

**Published:** 2024-12-05

**Authors:** Kella L Vangsness, Lillian Eklof, Savannah Moore, Todd Coffey, Jessica Evans, Richard Sloan

**Affiliations:** 1 General Surgery, Community Memorial Hospital, Ventura, USA; 2 Research, Idaho College of Osteopathic Medicine, Meridian, USA; 3 Microbiology, Idaho College of Osteopathic Medicine, Meridian, USA; 4 Osteopathic Medicine, Idaho College of Osteopathic Medicine, Meridian, USA

**Keywords:** attitudes, covid-19 vaccine, medical education curriculum, medical students, preclinical curriculum, vaccine education, vaccine hesitancy

## Abstract

Objective: The aim of this study is to investigate how demographic factors influence medical students' attitudes toward COVID-19 vaccines and their perceptions of vaccine education in medical school curricula.

Methods: A 28-question anonymous online survey was distributed to 640 medical students at one academic medical institution. Individual attitudes toward vaccines were evaluated using a 5-point Likert scale. Responses were tested for association with various demographic factors using the Chi-square test or Fisher's exact test.

Results: Two-hundred and forty-four responses out of 640 total surveyed revealed that 97.9% of the students had received at least one COVID-19 vaccine, 68.44% supported mandatory vaccinations, 16.81% did not believe vaccination protects others from getting sick, and 66.4% supported vaccine personal choice, with men showing a statistically stronger belief than women (p=0.0046). Approximately 25.1% of the students reported not receiving sufficient vaccine education in medical school and only 12.4% of all students considered medical school curricula as their most trusted source of vaccine information. Moreover, 8.6% of the students would not encourage patients to receive the COVID-19 vaccine and 8.2% were uncomfortable discussing patients' concerns. Unvaccinated students were more likely to not encourage patients to receive the vaccine (p<0.0001) and were more inclined to believe that acquiring natural immunity was preferable to vaccination (p<0.0001). One hundred percent of very conservative students, 97.8% of slightly conservative students, and students associated with any religion displayed statistical significance in endorsing vaccine personal choice (p=<0.0001), particularly among Christians (p=<0.0001). In contrast, 28% of very liberal and 48.3% of slightly liberal students agreed (p<0.0001). Additionally, the majority of liberal students agreed vaccines prevent the spread of disease while only 75% of conservative, independent, and neutral students agreed (p<0.001).

Conclusion: Despite high compliance, this cohort significantly demonstrated concern toward the COVID-19 vaccine, particularly those who were unvaccinated, had conservative political associations, and belonged to certain religious groups. These findings suggest identifying factors that impede medical education and an understanding of vaccines in order to improve physician training. We recommend an expanded medical curriculum to address these issues.

## Introduction

COVID-19 caused a heavy burden worldwide prompting lockdowns, masking, and social distancing measures [1]. These approaches were met with resistance due to fear of overriding individual autonomy, yet they alone are not enough to decrease morbidity and mortality without the help of vaccines [2,3]. At the time of data collection in April 2022, three COVID-19 vaccines were authorized for use in the United States. Only about 60% of the U.S. population were fully vaccinated with vaccine-hesitant individuals presenting a hurdle to increasing vaccine coverage [4,5]. Vaccine hesitancy refers to a reluctance to be vaccinated, even with good evidence of its safety and efficacy [6]. It has been identified by the World Health Organization as one of the top 10 threats to global health [2,6,7].

Unfortunately, healthcare providers are not immune to this phenomenon with high rates of COVID-19 vaccine hesitancy documented at an average rate of 29% globally and 44% within the U.S. [2,8-11]. Self-reported rationales for vaccine hesitancy include rapidity of vaccine development, rushed clinical trials, side effects, doubts about efficacy, concern over political motives, belief that COVID-19 is not real or not a serious problem, and distrust of the medical system in general [6,8,10-12]. Demographics linked with the highest rates of vaccine support include physicians who work directly with COVID-19-infected patients in a specialty with increased exposure, male gender, older age, higher education level, and previous vaccine receipt [2].

A single medical school institution reported that nearly one-quarter of students were hesitant to all vaccines while international COVID-19 vaccine hesitancy was calculated at a rate of 58%, all citing similar reasons for hesitancy [13-15].

Due to the importance of physician advocacy in obtaining high population vaccination rates, it is essential to understand the attitudes of medical students and the contributing factors of hesitancy toward the COVID-19 vaccines and developing vaccines [13-15]. This study seeks to investigate the influence of demographic factors on medical students' attitudes toward COVID-19 vaccines and their perceptions of vaccine education in medical school curriculum. Understanding the prevalence, predictive factors, and reasons for hesitancy among this cohort will help guide improved curricular changes to support the acceptance of vaccines.

## Materials and methods

The target population was one medical institution, Idaho College of Osteopathic Medicine. All 640 students currently enrolled during April 2022 were included. Survey creation, distribution, and collection were done through SurveyMonkey (SurveyMonkey Inc., Santa Mateo, U.S). An anonymous 28-question survey was created using newly written and validated items as well as questions obtained from previously validated vaccine hesitancy surveys [16,17]. Seventeen questions were used to assess attitudes, categorized into "safety", "efficacy", "professional responsibility", "education", and "mandates", with responses on a 5-point Likert scale ranging from "strongly agree" to "strongly disagree" (Table 1). Additionally, four "yes" or "no" questions were collected on personal COVID-19 vaccination, school requirements, caring for a COVID-19 patient, and trusted sources of vaccine information. Participants were also given a free response option for listing other trusted sources of vaccine information. Demographic information collected included gender identification, religious affiliation, political viewpoint, hometown population, medical school city population, and year in medical school. Free response options were provided for religious and political affiliation if "Other" was selected. Participants were also given the opportunity to share any further opinions about the COVID-19 vaccines (Supplemental Data, Table 5).

**Table 1:** Survey questions.

Category	Questions/Statements	Response Options
Agree/Disagree Questions	At least one of the COVID-19 vaccines is safe.	1 = Strongly Disagree, 2 = Slightly Disagree, 3 = Slightly Agree, 4 = Strongly Agree
	I am concerned about the side effects of the COVID-19 vaccines.	1 = Strongly Disagree, 2 = Slightly Disagree, 3 = Slightly Agree, 4 = Strongly Agree
	At least one of the COVID-19 vaccines is effective at preventing death or severe disease.	1 = Strongly Disagree, 2 = Slightly Disagree, 3 = Slightly Agree, 4 = Strongly Agree
	I am concerned about the rapid development and FDA approval of at least one of the COVID-19 vaccines.	1 = Strongly Disagree, 2 = Slightly Disagree, 3 = Slightly Agree, 4 = Strongly Agree
	The vaccines are necessary because COVID-19 is a serious threat.	1 = Strongly Disagree, 2 = Slightly Disagree, 3 = Slightly Agree, 4 = Strongly Agree
	It is better to develop natural immunity to COVID-19 than to get vaccinated.	1 = Strongly Disagree, 2 = Slightly Disagree, 3 = Slightly Agree, 4 = Strongly Agree
	As a future physician, I must learn about the safety and efficacy of vaccines for myself and my patients.	1 = Strongly Disagree, 2 = Slightly Disagree, 3 = Slightly Agree, 4 = Strongly Agree
	I have received sufficient education about vaccines and their safety in medical school.	1 = Strongly Disagree, 2 = Slightly Disagree, 3 = Slightly Agree, 4 = Strongly Agree
	It is my role as a future physician to question the safety and efficacy of the COVID-19 vaccines.	1 = Strongly Disagree, 2 = Slightly Disagree, 3 = Slightly Agree, 4 = Strongly Agree
	I would not encourage patients to get the COVID-19 vaccine.	1 = Strongly Disagree, 2 = Slightly Disagree, 3 = Slightly Agree, 4 = Strongly Agree
	I feel comfortable discussing patients’ concerns about the COVID-19 vaccines.	1 = Strongly Disagree, 2 = Slightly Disagree, 3 = Slightly Agree, 4 = Strongly Agree
	The government should not set mandates with respect to COVID-19 vaccinations.	1 = Strongly Disagree, 2 = Slightly Disagree, 3 = Slightly Agree, 4 = Strongly Agree
	The COVID-19 vaccination should be mandatory for all medical students.	1 = Strongly Disagree, 2 = Slightly Disagree, 3 = Slightly Agree, 4 = Strongly Agree
	The only reason I will get a COVID-19 vaccine is if it is mandated by health systems/medical school.	1 = Strongly Disagree, 2 = Slightly Disagree, 3 = Slightly Agree, 4 = Strongly Agree
	To protect public health, we should follow government guidelines about the COVID-19 vaccine.	1 = Strongly Disagree, 2 = Slightly Disagree, 3 = Slightly Agree, 4 = Strongly Agree
	Being vaccinated against COVID-19 helps protect other people from getting sick.	1 = Strongly Disagree, 2 = Slightly Disagree, 3 = Slightly Agree, 4 = Strongly Agree
	COVID-19 vaccination should be a personal choice.	1 = Strongly Disagree, 2 = Slightly Disagree, 3 = Slightly Agree, 4 = Strongly Agree
Yes/No Questions	I have received at least one dose of the COVID-19 vaccine.	Yes / No
	I am required to receive the COVID-19 vaccine by my school or clinical site.	Yes / No
	I care for or have cared for COVID-19 patients during medical school.	Yes / No
My most trusted source of vaccine information is:	My most trusted source of vaccine information is:	Medical school curricula, Public health organizations, Medical literature, News and media, Social media, Friends/family, Other (fill in)
Demographics	What is your program?	M.D. / D.O.
	What is your current year of medical education?	OMS/MS-I, OMS/MS-II, OMS/MS-III, OMS/MS-IV
	Location of medical school	Large Urban (1,000,000+), City (250,000-999,999), Suburban (25,000-250,000), Rural (<25,000), Prefer not to answer
	Size of hometown	Large Urban (1,000,000+), City (250,000-999,999), Suburban (25,000-250,000), Rural (<25,000), Prefer not to answer
	Political viewpoint	Very conservative, Slightly conservative, Neutral, Slightly liberal, Very liberal, Independent, Other, Prefer not to answer
	Religion	Atheist, Agnostic, Ancient religion, Buddhist, Christian, Hindu, Jehovah’s Witness, Jewish, Latter-day Saints, Muslim, Orthodox-Greek, Orthodox-Russian, Protestant, Roman Catholic, Sikh, Traditional/Folk religion, Not religious, Other, Prefer not to answer
	Gender	Female, Male, Non-Binary, Transgender, Self-describe, Prefer not to answer
Free Response	Further opinions about COVID-19 vaccines	Open-ended

The survey (Table 1) was validated by an expert committee of three medical professionals on institutional faculty to assess face validity, content validity, and internal consistency. No authors contributing to data analysis or reporting were included on the validating panel. Students were contacted through medical school email addresses. They were notified of the survey and provided with a link to the informed consent and survey with one reminder email. Survey construction, distribution, and collection were completed through Survey Monkey over a two-week period in April 2022.

Exempt IRB approval was obtained through Boise State University (Protocol #998-SB22-038). Boise State University serves as the Institutional Review Board of record and is utilized by Idaho College of Osteopathic Medicine. Individual attitudes and student beliefs about vaccines were evaluated for association with various demographic factors using the Chi-square test or Fisher's exact test. Chi-square analysis was performed comparing Yes and No, as well as comparing agree (strongly agree and agree) versus disagree (strongly disagree and disagree). The significance level is set at <0.05. Study participants were required to be students enrolled at medical colleges in the United States and included both preclinical and clinical students. There were no exclusion criteria. The Consensus-Based Checklist for Reporting of Survey Studies (CROSS) was used to assist in development and writing [18].

Only one respondent listed "non-binary" for gender and was removed from the data set so as to protect identification. Five students chose the gender option "prefer not to answer" and thus were not included in gender analysis. Hometown and school location demographics were additionally removed due to students incorrectly identifying the population size of the institution’s location and therefore hometown size could be incorrectly reported.

## Results

Of the 640 medical students surveyed, 244 (37.97%) responses were collected. Among them, 146 (60.1%) were pre-clinical students, with 31.97% in their first year (MS-I) and 27.87% in their second year (MS-II). The remaining 97 (40.16%) were clinical students, with 20.49% in their third year (MS-III) and 19.67% in their final year (MS-IV). Gender distribution was 41.80% female, 55.74% male, 0.41% non-binary, 1.23% preferred not to answer, and 0.82% self-described (Table 2). Participant responses are reported by grouping “strongly agree” with “agree”, and “strongly disagree” with “disagree”, into unified “agree” and “disagree” terms for interpretation unless specified. Free responses are included in Supplemental Data Table 5.

**Table 2:** Demographic characteristics of participants (N=243). Note*:* Statistical parameters are represented as frequency (N) and percent (%).

Demographic Variables	N (%)
Gender	
Male	136 (55.74%)
Female	102 (41.80%)
Non-binary/Prefer not to answer	6 (2.50%)
Year of education	
First year	78 (31.97%)
Second year	68 (27.87%)
Third year	50 (20.49%)
Fourth year	48 (19.67%)
Religious affiliation	
Atheist	27 (11.11%)
Agnostic	31 (12.76%)
Christian	48 (19.75%)
Hindu	4 (1.65%)
Jewish	2 (0.82%)
Latter-day Saints	41 (16.87%)
Muslim	7 (2.88%)
Greek Orthodox	1 (0.41%)
Protestant	5 (2.06%)
Roman Catholic	23 (9.47%)
Sikh	1 (0.41%)
Not religious	32 (13.17%)
Preferred not to answer	13 (5.35%)
Other	8 (3.29%)
Political affiliation	
Very conservative	20 (8.20%)
Slightly conservative	45 (18.44%)
Politically neutral	32 (13.11%)
Slightly liberal	60 (24.59%)
Very liberal	45 (18.85%)
Independent	28 (11.48%)
Other	8 (3.28%)
Prefer not to answer	5 (2.06%)

Religious and political affiliation

Religious beliefs varied with Latter-day Saints (16.87%), Christian (19.75%), and Agnostic (12.76%) being the most represented groups followed by Atheists (11.11%) and students who identified as not religious (13.17%). Politically, 24.59% identified as slightly liberal, 18.85% as very liberal, and 13.11% as neutral. Conservative groups made up 26.64% of the respondents (8.20% very conservative, 18.44% slightly conservative), with 11.48% identifying as independent (Table 2).

Vaccination status and government mandates

Nearly all students (97.94%) had received at least one COVID-19 vaccine dose with 66.26% reporting vaccination was required by their school or clinical site; 51.44% of students reported they had not cared for a COVID-19 patient during medical school (Table 3). The majority (66.4%) believed vaccination should remain a personal choice, with support for this idea strongest among men and those with religious affiliations (p < 0.01). Political views also influenced opinions with 100% of very conservative students supporting personal choice compared to 28% of very liberal students.

**Table 3:** Personal vaccination and experiences. Note: Statistical parameters are represented as frequency (N) and percent (%). The significance level is set at <0.05 for the two-proportion Z-test.

	Yes N (%)	No N (%)	p-value
Personal vaccination behavior			
I have received at least one dose of the COVID-19 vaccine	238 (97.94)	5 (2.06)	<0.001
I am required to receive the COVID-19 vaccine by my school or clinical site	161 (66.26)	82 (33.74)	<0.01
Experience with COVID-19			
I care for or have cared for COVID-19 patients during medical school	118 (48.56)	125 (51.44)	0.67

Trust in vaccine information sources

Medical literature was the most trusted source of vaccine information (60.08%) followed by public health organizations (21.81%) and medical school resources (12.4%). Students who relied on “other” sources of information were more likely to question vaccine safety and efficacy.

Concerns about vaccine safety and development

Most students (95%) believed that at least one COVID-19 vaccine was safe (Table 4). However, concerns about side effects were prevalent among those unvaccinated (p <0.001), conservatives, and independents. Political affiliation strongly influenced these concerns, with 55% of very conservative and 44.44% of slightly conservative students expressing apprehensions. Protestant (60%), Roman Catholic (41.67%), and Latter-day Saint (44.19%) students reported higher concern levels compared to Atheists (10.71%) and Agnostics (29.03%). Concerns also extended to the speed of vaccine development and FDA approval with 36.07% expressing unease. Politically conservative and independent students were significantly more likely to report these concerns (p < 0.001). Religious affiliation further shaped responses with Latter-day Saints (55.81%) and Protestants (60%) being the most concerned about speed of development compared to Muslims (14.29%) and Atheists (17.86%).

**Table 4:** Survey responses by category. Note*:* Statistical parameters are represented as frequency (N) and percent (%). Chi-square analysis was performed comparing agree (strongly agree and agree) versus disagree (strongly disagree and disagree). The significance level is set at <0.05.

Survey Item	Participant Responses					
	Agree		Disagree			
	Strongly Agree N (%)	Agree N (%)	Disagree N (%)	Strongly Disagree N (%)	p-Value	
Safety						
At least one of the COVID-19 vaccines is safe	166 (86.03)	67 (27.46)	10 (4.10)	166 (68.03)	<0.01	
I am concerned about side effects from the COVID-19 vaccines	22 (9.02)	59 (24.18)	103 (42.21)	60 (24.59)	<0.01	
I am concerned about the rapidity of the development and FDA approval of at least one of the COVID-19 vaccines	28 (11.48)	60 (24.59)	86 (35.25)	70 (28.69)	<0.01	
Efficacy						
At least one of the COVID-19 vaccines is effective at preventing death or severe disease	170 (69.97)	57 (23.36)	13 (5.33)	4 (1.64)	<0.001	
The vaccines are necessary because COVID-19 is a serious threat	136 (55.74)	66 (27.05)	32 (13.11)	10 (4.10)	<0.001	
It is better to develop natural immunity to COVID-19 than to get vaccinated	13 (5.33)	37 (15.16)	93 (38.11)	101 (41.39)	<0.01	
Professional Responsibility						
It is my role as a future physician to learn about the safety and efficacy of vaccines in general for myself and my patients	217 (88.93)	25 (10.25)	0 (0)	2 (0.82)	<0.001	
It is my role as a future physician to question the safety and efficacy of the COVID-19 vaccines	136 (55.74)	86 (35.25)	19 (7.79)	3 (1.23)	<0.01	
I would not encourage patients to get the COVID-19 vaccine	9 (3.69)	12 (4.92)	56 (22.95)	167 (68.44)	<0.01	
I feel comfortable discussing patients’ concerns about the COVID-19 vaccines	97 (39.75)	127 (52.05)	19 (7.79)	1 (0.41)	<0.01	
To protect public health, we should follow government guidelines about the COVID-19 vaccine	91 (37.30)	108 (44.26)	30 (12.30)	15 (6.15)	<0.01	
Being vaccinated against COVID-19 helps protect other people from getting sick	144 (59.02)	59 (24.18)	26 (10.66)	15 (6.15)	<0.01	
Education						
In general, I have received sufficient education about vaccines and their safety in medical school	64 (26.23)	118 (48.36)	51 (20.90)	11 (4.51)	<0.01	
Mandates						
The government should not set mandates with respect to COVID-19 vaccinations	66 (27.05)	51 (20.90)	78 (31.97)	49 (20.08)	<0.01	
The COVID-19 vaccination should be mandatory for all medical students	109 (44.67)	58 (23.77)	47 (19.26)	30 (12.30)	<0.01	
The only reason I will get a COVID-19 vaccine is if it is mandated by health systems/medical school	14 (5.74)	19 (7.79)	58 (23.77)	153 (62.70)	<0.01	
Being vaccinated against COVID-19 helps protect other people from getting sick	144 (59.02)	24.18 (59)	26 (10.66)	15 (6.15)	<0.01	
COVID-19 vaccination should be a personal choice	75 (30.74)	87 (35.66)	59 (24.18)	23 (9.43)	<0.01	

Perceptions of vaccine efficacy

While the majority believed vaccines prevented severe disease or death, 7% disagreed with this statement, particularly among unvaccinated students (p < 0.01). Political affiliation played a notable role with 20% of very conservative students and 17.86% of independents expressing doubts about vaccine efficacy. Latter-day Saints (20.93%) and Christians (8.33%) were the least likely to believe in vaccines' effectiveness, although this finding was not statistically significant.

Natural immunity and COVID-19 as a threat

Around 20.49% of students believed that natural immunity was preferable to vaccination. Unvaccinated students (p < 0.01), men, and those who trusted “other” sources of information were more inclined to support this idea. Political beliefs were strongly associated with 55% of very conservative students supporting natural immunity compared to just 2.17% of very liberal students. Additionally, 17.21% of respondents did not consider COVID-19 a serious threat, with significant political divides. Only 55% of very conservative students believed that vaccination was necessary to address COVID-19 compared to 100% of slightly liberal students (p < 0.01).

Professional responsibility and vaccine education

An overwhelming majority of students (99.18%) believed learning about vaccine safety and efficacy was part of their role as future physicians (p <0.001). However, 9.05% disagreed that it was their responsibility to question vaccine efficacy. Of the respondents, 25.1% felt they had not received sufficient education on vaccines in medical school, although vaccinated students were more likely to feel adequately informed (p <0.001).

Attitudes toward government guidelines and mandates

Students' opinions on government guidelines were divided along political lines. All very liberal (100%) and slightly liberal (98.33%) students agreed with following government health guidelines while only 45% of very conservative students and 50% of those identifying as "Other" agreed (p < 0.0001). Similarly, religion influenced these views with 100% of Muslim students agreeing with following government mandates compared to 48.84% of Latter-day Saints. Support for mandatory vaccination also varied. Atheists (92.86%) and Agnostics (83.87%) were among the strongest supporters of mandates, while only 39.53% of Latter-day Saints and 62% of Christians agreed. Additionally, 59.26% of students believed the government should not be involved in setting vaccination mandates, with conservative and independent students strongly opposing government involvement (p < 0.01).

Comfort discussing vaccines with patients

Only 8.2% of students reported discomfort in discussing patient concerns about the COVID-19 vaccine. Those with experience caring for COVID-19 patients were more comfortable having these conversations. There were no significant differences based on students’ year in school, religion, or political affiliation.

## Discussion

This study investigated medical students' attitudes toward the COVID-19 vaccine and the inclusion of vaccine education in medical school curricula. Results revealed that although most students were vaccinated against COVID-19, many expressed reluctance to encourage vaccination among patients or felt uneasy addressing patient concerns about the vaccine. However, there was unanimous agreement on believing they are responsible as future physicians to learn about and critically evaluate vaccine safety and efficacy.

Despite this, a quarter of students reported receiving inadequate education. One national study found that while more than half of students supported mandatory vaccination for healthcare providers, 23% exhibited vaccine hesitancy [19,20]. As more individuals turn to social media and news outlets for health information it is essential to incorporate strategies for engaging and educating patients about vaccine safety early in medical training [13,19,20]. Additionally, about 7% of students doubted the effectiveness of at least one vaccine in preventing serious diseases, and 16.9% questioned whether vaccination protects others from illness. Research suggests a correlation between robust vaccine knowledge and higher vaccination rates with less informed students being less likely to receive vaccines themselves [21,22-24].

A staggering number of students (83%) reported not receiving adequate education on vaccines during their medical school education despite the majority believing it is their role to learn about and question safety and efficacy in their role as a physician. Only 12.4% listed medical school curricula as their most trusted source of vaccine information. Perhaps a robust curriculum addressing vaccine hesitancy may improve comfortability while discussing vaccines with patients. Potential impacts of vaccine hesitancy among healthcare professionals will create an expanding barrier for public health support and disease control, not only for COVID-19, but also for additionally helpful vaccines such as HPV, MMR, and Tdap.

This data found that 33% expressed concerns over side effects from the COVID-19 vaccines and 66.7% supported vaccine personal choice, similar to data found within the medical community [13,19-21]. Understanding medical student attitudes and factors contributing to hesitancy will allow for tailored education and counseling to improve student understanding and, just as importantly, provide adequate tools to support critical thinking and interpretation of data. Doing so will better equip physicians to tackle patient hesitancy. Unprepared students will create ill-equipped physicians and, in turn, leave patients unprotected or without a healthcare provider who can properly discuss vaccine benefits. If patients do not engage with providers they can turn to voices outside of healthcare and risk trusting those who do not operate with patient protection in mind [20,21]. Trusting “Other” sources of information and a low rate of valuing medical school curricula may point to areas of improvement within schools.

Identified factors correlated with vaccine hesitancy were certain political and religious affiliations. For example, those who identified as conservative were less likely to support vaccines for COVID-19 because they did not believe it to be a severe threat compared to students who identified as liberals. There were also differences in attitudes regarding vaccines based on religious affiliations; for example, 69.8% of Latter-day Saints and 75% of Christians regarded at least one of the vaccines to be effective at averting death or severe disease, compared to Muslims, Roman Catholic, Protestant, or Other. Conservative students and association with any religion displayed statistical significance in endorsing vaccination as a personal choice, whereas only 28% of liberal students agreed. The majority of liberal students believed that vaccines prevent disease spread, while only 75% of conservative, independent, and politically neutral students agreed. The role of political association and religious beliefs have been noted to influence vaccine opinion and this data corroborates existing theories of vaccine hesitancy models [22,24-26]. Students more likely to request the COVID-19 vaccine were likelier to trust public health experts with accompanying leanings towards less hesitancy for any side effects or vaccine mandates [19,23]. It is not surprising these discrepancies among political and religious leanings are seen within this cohort, as they are well established within the literature [22,24-27]. Unvaccinated students were significantly more likely to express concerns over side effects; more inclined to believe that acquiring natural immunity was preferable to vaccination and were more likely not to encourage patients to receive the vaccine.

When considering the relevance between education and medical care, understanding the narratives within these communities and addressing them within medical education will allow providers to establish an improved rapport with patients and have a greater understanding of patient desires and ways to engage in meaningful discussion on vaccines [27].

Limitations

Limitations present in this study include selection bias from one medical school, which is not representative of all medical education and opinions. This was an optional survey, naturally creating a bias for those who chose to respond. Attitudes and beliefs surrounding hesitancy were not comprehensively studied, nor were all demographics, such as age or race/ethnicity, possibly leaving gaps within the findings. There were limitations in reporting hometowns, as witnessed by multiple reports of the size of the school town, varying from rural to urban (the school city size is 500,000). This made the data unreliable and therefore, they were excluded.

Future considerations

Recommendations for future research include a more robust collection among other medical school cohorts and demographic variables. Future exploration should inquire more specifically about vaccine education and gaps within the data to more comprehensively understand current limitations within education and reasons for hesitancy with patient counseling.

## Conclusions

The study found that there were significant levels of vaccine hesitancy among this group of medical students. Those with conservative political affiliations and certain religious groups were less supportive of COVID-19 vaccination despite being highly compliant with self-vaccination. Identifying the factors contributing to these misconceptions is important for overcoming barriers to proper vaccine education and improving attitudes. It is crucial for physicians to be able to have open and comfortable discussions with patients about vaccinations and reasons for hesitancy in order to improve patient outcomes and reduce morbidity and mortality from COVID-19. Enhancing undergraduate medical education by including a comprehensive curriculum that addresses vaccine development, distribution, and hesitancy is recommended.

## Appendices

**Table 5:** Qualitative data from the survey. Note:* *Quotes were derived from the free text responses in the survey.

Student Narrative Responses
I have concerns regarding the expeditious approvals of boosters, particularly in young people, where GOOD (non-antibody titer) data is lacking. I believe that evidence should guide decision-making, and when it does not, I grow to have concerns. This is especially true when the public health and CDC fail to recognize nuance in their decision-making. Randomized control trials with outcome measures such as hospitalizations and mild/severe disease are helpful; antibody titers are just one facet of immunity and, in my opinion, should not be used solely for decision-making. In addition, I am skeptical about the idea of a company that makes the COVID-19 vaccines being the one that determines that we need more through press releases and memos.
I do not believe anyone should be mandated to receive this vaccine. Not almost everything you hear about the vaccine from any new source even agrees with what data on the CDC website says about its effectiveness and safety. Now, do I think it can be beneficial for a certain population? Of course, I do, and if someone wants to get it, that is great, but strictly speaking, from looking at the data from the CDC, I am more likely to develop serious SE from the vaccine than from actually having COVID-19. Therefore, to be told that I must get it or I cannot finish medical school is frustrating. Plenty of people already distrust the medical system, and what we have done in the last two years has made it even worse. and to be honest, I would have thought twice before even going into medicine if I had seen this level of corruption before starting; that would have been wrong, though, because people like me are going to make big changes in medicine that are going to really focus on helping people and creating a trust between the population and medical professionals. It will take a lot of work, though, thanks to pushy politicians and news outlets that are being paid under the table to push this stuff.
It is unfortunate to have accepted and forced the widespread administration of such vaccines while not following the protocols of years before. The lightning-speed production has, in fact, resulted in a multitude of side effects, which, for many, have caused a medical illness that supersedes the effects of contracting the virus naturally. I am happy to receive general vaccines, which have time to back their efficacy and side effect profile.
I should’ve invested more money in modern
I think the biggest shortcoming of COVID-19 vaccines has been the governments' highly suggestive, if not outright, wording that the COVID-19 vaccine is 100% effective and a “magic pill of sorts.” Had they been more transparent that this is a vaccine with moderate efficacy, not an immunization, we as a society would be far more aligned.
I think the vaccine is a good idea for most people, and I would never encourage anyone from getting it, but I think the heavy push from the government did more harm than good
I agree with the science behind it, but I believe it should be a choice, not a mandate, for someone to do something.
the only hesitancy I have with the COVID vaccines is because they were tested on cell lines derived from aborted fetal cells; if there were no link, I would not have hesitancy
With omicron and its high breakthrough rate, the vaccine seems to be mostly effective at preventing severe disease and less effective at reducing transmission. In addition, the survey did not ask if I would have been vaccinated had it not been mandated by my school. The answer is yes
The question regarding which is “better” is poorly worded & feels divisive… I strongly agree that natural immunity is more effective/protective than vaccination without exposure. However, that does not negate the value of the vaccine.
The questions about vaccine recommendations are overly broad. Vaccines are a type of medicine. There are no medicines that are risk free, and no medicine is useful in every individual.
I am vaccinated but refuse to get the booster based on the lack of knowledge regarding when to get it and how efficacious it is.
If you are going into the healthcare profession, there should be an understanding that vaccines will likely be required.
Personal choice from individual investigation must determine vaccination
Each person's case should be considered individually. Mandates from the government or businesses interfere with the provider/individual decision-making, which is best for people's health
It is more anti-mandate from a political standpoint than from a medical one.
I find the lack of a neutral option in the Likert scale limits the usefulness of this survey. These questions also seem leading in nature.
Many of these questions are poorly worded and unclear as to exactly what they are asking
I believe that as members of the healthcare team, it is our duty to ensure public health and the safety of our patients. It is known that unvaccinated individuals who have been infected with COVID-19 are much more likely to utilize healthcare resources. Covid vaccines decrease this utilization, allowing for those resources to be available for other needs (ex, car accidents, trauma, MIs, strokes, etc. are still happening, and when hospitals are overwhelmed, we do not have the resources to accommodate appropriate care and resources to all). Additionally, being double vaccinated and getting Covid twice in 2 months, with the second infection being more severe, made me realize how young people are dying who have been previously healthy. Having also been dealing with this long Covid since I cannot imagine how much more severe it would be and how much more healthcare resources I would have to utilize. There are serious effects and impacts on the healthcare system and public health with lower vaccination rates. I believe mandated vaccines should be required if one wants to participate in public life.
I am actually neutral on the safety of the COVID-19 vaccines. I do think they are beneficial to some populations, such as those with comorbidities. I think they may be more of a risk in young patients. We also have not really been taught about mRNA vaccines in medical school. I think in 10-20 years, we will have a better understanding of the safety and efficacy of these vaccines. There are still a lot of grey areas in the research.
We should have a lecture on the common reasons for vaccine hesitancy and what the research says about those reasons.
I am not sure what the threshold for immunity is for the COVID-19 vaccine, especially since you can still catch COVID-19 while vaccinated. So I don’t see how everyone receiving it will protect other people if there isn’t necessarily a herd immunity threshold besides lowering everyone’s chances of catching it
*Vaccination should be required for all medical students unless they have a medical exemption.
If medical students or physicians do not believe in the efficacy and safety of the COVID-19 vaccines and the majority of vaccines in general, perhaps they should go back to undergrad and pay better attention to microbiology, biochemistry, and genetics. Either that or pick a different profession. I do not believe that the government should be able to mandate vaccinations for the public, but both government and private employers should reserve the right to require vaccinations, as well as schools. For those who feel frustrated by this, try being a woman who needs an abortion in Texas. Wild concept about personal choice and freedom.
Governments should not issue mandates requiring the vaccine, as personal choice is valuable, but businesses and organizations are 100% within their right to mandate it. True religious exemptions due to going against faith are also very rare; if supposedly it is due to using stem cells from aborted fetuses, then they should also refrain from several other medicines like Tylenol.
Healthcare should always be a choice, even when patients make the "wrong choice"
Public Health is not a personal choice, and opting out of the vaccine should only be due to a medical reason. The COVID-19 vaccine should be treated as all other lifesaving vaccinations and should not be up for political debate.
